# Extraction Optimization, Structural Characterization, and Antioxidant Activities of Polysaccharides from Cassia Seed (*Cassia obtusifolia*)

**DOI:** 10.3390/molecules24152817

**Published:** 2019-08-02

**Authors:** Ding-Tao Wu, Wen Liu, Qiao-Hong Han, Ping Wang, Xian-Rong Xiang, Ye Ding, Li Zhao, Qing Zhang, Su-Qing Li, Wen Qin

**Affiliations:** Institute of Food Processing and Safety, College of Food Science, Sichuan Agricultural University, Ya’an 625014, China

**Keywords:** *Cassia obtusifolia*, polysaccharides, microwave-assisted extraction, chemical structure, antioxidant activity

## Abstract

In order to explore Cassia seed polysaccharides (CSPs) as natural antioxidants for application in the functional-food industry, microwave-assisted extraction (MAE) was optimized for the extraction of CSPs by using a response surface methodology. Furthermore, the chemical structures and antioxidant activities of CSPs extracted by MAE and hot water extraction were investigated and compared. The maximum extraction yield of CSPs extracted by MAE (8.02 ± 0.19%) was obtained at the optimized extraction parameters as follows: microwave power (415 W), extraction time (7.0 min), and ratio of water to raw material (51 mL/g). Additionally, the contents of the uronic acids, molecular weight, ratio of constituent monosaccharides, intrinsic viscosities, and degrees of esterification of CSPs were significantly affected by the MAE method. Moreover, CSPs exhibited remarkable 2,2′-azino-bis(3-ethylbenzothiazoline-6-sulfonic acid) ABTS, 2,2-diphenyl-1-(2,4,6-trinitrophenyl) hydrazyl DPPH, nitric oxide, and hydroxyl radical scavenging activities as well as reducing power. The high antioxidant activities observed in CSPs extracted by MAE could be partially attributed to its low molecular weights and high content of unmethylated galacturonic acid. Results indicate that the MAE method could be an efficient technique for the extraction of CSPs with high antioxidant activity, and CSPs could be further explored as functional food ingredients.

## 1. Introduction

Oxidative stress is usually caused by reactive oxygen species (ROSs) produced during physiologic events [1]. It is well known that some of these ROSs play positive roles in vivo [2]. However, an excessive amount of ROSs can damage cellular components such as lipids, proteins, and DNA when the innate defense in the human body is not enough for severe oxidative stress, a variety of diseases will happen including cancer, aging, and other diseases [3,4,5]. Generally, antioxidants play important roles in the protection of living organisms [6]. Many antioxidants have been found to be very important in reducing oxidation damage in recent years, but many synthetic antioxidants have toxicological reports [7]. Hence, the search for low toxicity or nontoxic natural antioxidants from plants such as plant derived polysaccharides has become a research hotspot [8]. 

*Cassia obtusifolia* L. is a plant of the Leguminosae family, which is a small to medium size tree that is native to tropical and Central America and West India, and widespread in Brazil, a tropical country [9,10]. The dry and ripe seed of Cassia is well known as ‘Jue Ming Zi’ in Chinese [11]. Previous studies have indicated that the Cassia seed exhibits health beneficial effects [12] such as lowering blood glucose level [12], antiallergic activity [13], and antioxidant activity, which is the effect of most concern [14,15]. Phenolic compounds in ethanol extracts of Cassia seed have been identified as natural antioxidants in Cassia seed [12,14,15]. In addition, polysaccharides are also considered to be the main bioactive components in Cassia seed [11,16,17,18,19], and possess multiple bioactivities such as remarkable antioxidant activity [2], immunomodulatory activity [11], α-amylase inhibitory effect [16], and anti-tumor activity [19]. Therefore, polysaccharides from the Cassia seed can be further explored as functional food ingredients for application in the functional-food industry.

In general, different extraction methods significantly influence the yields, physicochemical characteristics, and bioactivities of polysaccharides extracted from natural resources [20,21,22]. To extract the polysaccharides from plants, the traditional hot water extraction (HWE) is commonly used. Indeed, the HWE method has been optimized for the extraction of polysaccharides from Cassia seed, and the maximum extraction yield is obtained under the optimal conditions as follows: extraction temperature (80 °C), extraction time (3.5 h), and ratio of water to raw material (30 mL/g) [2]. The HWE method has some shortcomings of a longer extraction time and lower extraction efficiency when compared with other new techniques [2,23,24,25]. Additionally, some physical methods can be used to facilitate the extraction process. Microwave-assisted extraction (MAE) has several advantages during extraction such as a low operation time, high efficiency, and high extraction yield [26,27,28]. Indeed, several studies have demonstrated that natural polysaccharides extracted by MAE exhibit much higher antioxidant activity than that of HWE [21,29,30]. The microwave-assisted aqueous two-phase extraction has been utilized to extract the polysaccharides from Cassia seed [31], and the maximum extraction yield is obtained under the optimal conditions as follows: the composition of 25.4% ethanol and 22.0% ammonium sulfate for the aqueous two-phase system, temperature of 80 °C, 20 min extraction time, and a 60:1 solvent-to-material ratio. However, the composition and extraction time of this procedure is relatively complex and long. Furthermore, to the best of our knowledge, the microwave-assisted extraction of polysaccharides from Cassia seed have seldom been optimized, and the effects of MAE and HWE methods on the chemical characteristics and antioxidant activities of CSPs have seldom been compared. Therefore, in order to explore CSPs as functional food ingredients and natural antioxidants for industrial applications, and to evaluate the effects of the MAE method on the physicochemical characteristics and antioxidant activities of CSPs, the MAE method was first optimized for the extraction of CSPs by using the response surface methodology, and then the chemical structures and antioxidant activities of CSPs obtained by two kinds of extraction methods (MAE and HWE) were compared.

## 2. Results and Discussions

### 2.1. Optimization of Microwave-Assisted Extraction of Polysaccharides from Cassia Seed

**Figure 1:**
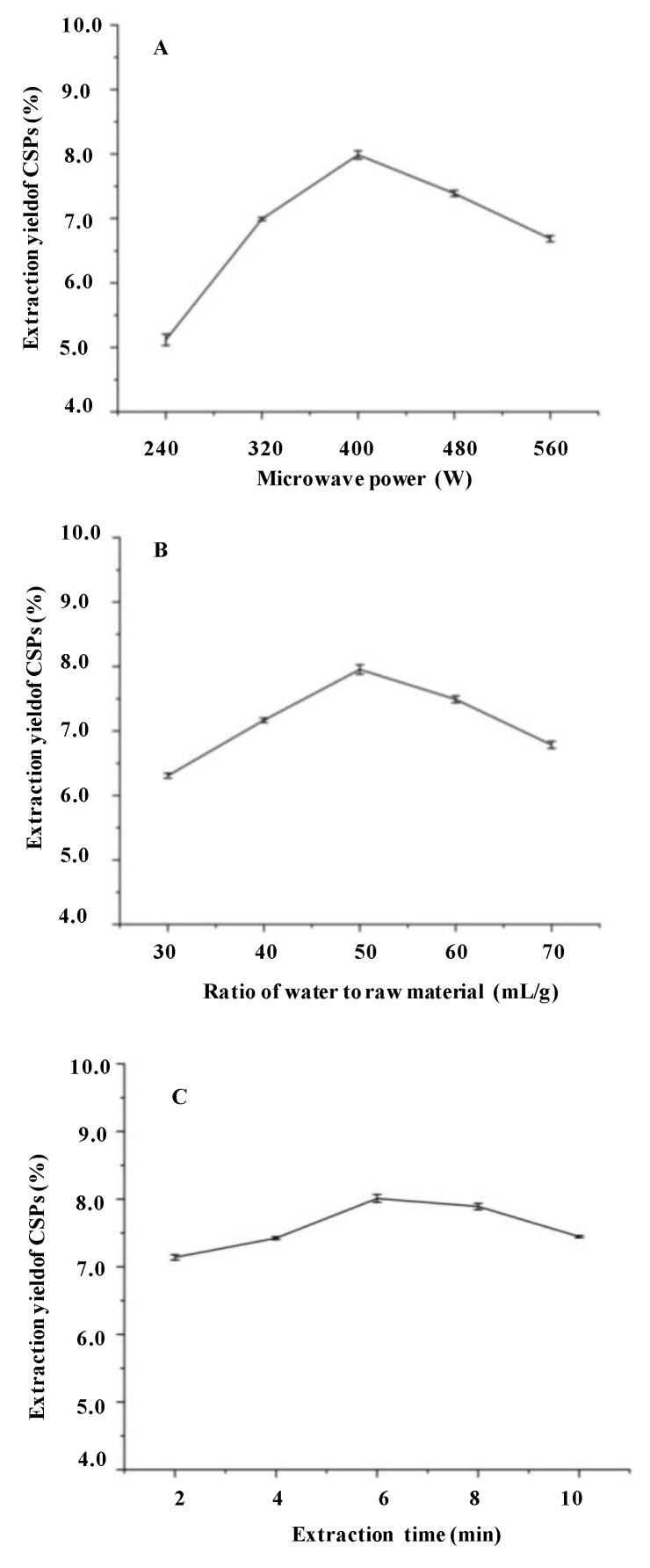
Effect of different microwave power (**A**), ratio of water to raw material (**B**), extraction time (**C**) on the extraction yields of CSPs.

The microwave power, microwave extraction time, and ratio of water to raw material are significant parameters that affect the extraction yield of polysaccharides [21]. The effects of these parameters on the yields of CSPs are shown in Figure 1. Briefly, the yields of CSPs were positively related to an increase of microwave power from 240 to 400 W, and then decreased (Figure 1A). This might be because an increase in microwave power could promote heat generation in the mixture and enhance the extraction yields. However, a too high microwave power could cause the degradation of polysaccharides [32]. In addition, the yields of CSPs increased while the ratio of water to raw material increased from 30 to 50 mL/g, and reached the highest yield at 50 mL/g (Figure 1B). However, the yield of CSPs decreased significantly while the ratio of water to raw material continued to increase, which might be due to a higher ratio of water to raw material, possibly leading to a lower density and viscosity, thereby facilitating the dilution of polysaccharides in the solvent [33]. Furthermore, the yields of CSPs increased while the extraction time increased from 2 to 6 min, and the highest extraction yield was reached at 6 min (Figure 1C). The yield decreased when the extraction time increased continuously. This could imply that an increased extraction time can make the extraction procession become easier and quicker, but the degradation of polysaccharides might be caused by a too long extraction time [34,35]. Finally, the optimal extraction conditions were determined to be 400 W, 50 mL/g, and 6 min. Based on the results above, a Box-Behnken experimental design (BBD) was further utilized to optimize the MAE conditions. Table 1 summarizes the BBD matrix and the experimental data for the MAE method. A final second-order polynomial equation in terms of coded values was obtained by applying multiple regression analysis:

$$\begin{array}{c} Y\;=\;+\;7.98\;+\;0.26X_{1}\;+\;0.13X_{2}\;+\;0.33X_{3}\;+\;0.076{\;X}_{1}X_{2}-0.15{\;X}_{1}X_{3}\\ +0.23X_{2}X_{3}-0.61X_{1}^{2}-0.74X_{2}^{2}-0.60X_{3}^{2} \end{array}$$

where Y represents the extraction yield, and X_1_, X_2_, and X_3_ are the microwave power, ratio of water to raw material, and extraction time, respectively.

In the BBD analysis, the significance of the model equation was checked by the *F*-values and *p*-value [21]. As shown in Table 2, the fitness of the model was highly significant based on the high *F*-value (227.16) and the very low *p*-value (*p* < 0.0001) [20]. Furthermore, the lack of fit was not significant based on the lack of fit *F*-value of 1.67 and *p*-value of 0.3099 (*p* > 0.05). Results indicated that the model equation was adequate for predicting the yield of CSPs [30]. Furthermore, this model had good precision and reliability according to the low value of the coefficient variation (C.V., 0.88%) and the high value of the adeq. precision (40.84) [23]. Furthermore, the R^2^ and R_adj_^2^ were 0.9966 and 0.9922, respectively, which indicated a high degree of correlation between the observed and predicted values [32]. In addition, the linear coefficients (X_1_, X_2_, X_3_), interaction coefficients (X_1_X_2_, X_1_X_3_, and X_2_X_3_), and quadratic term coefficients (X_1_^2^, X_2_^2^, and X_3_^2^) of the model equation were significant (*p* < 0.05), respectively.

**Figure 2:**
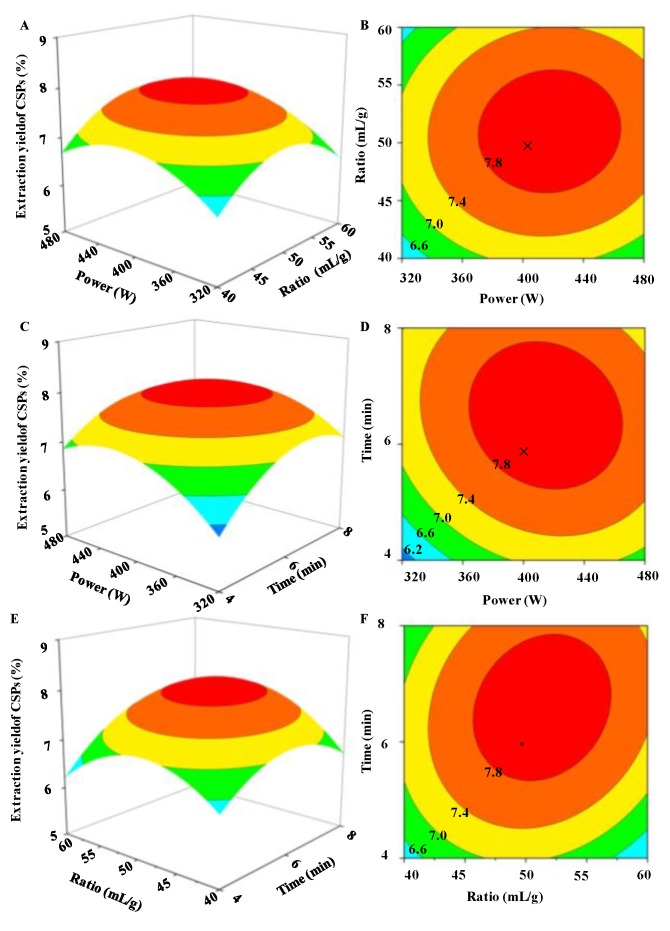
Three-dimensional response surface (left) and two-dimensional contour (right) plots of microwave-assisted extraction. (**A**,**B**) microwave power and ratio of water to raw material; (**C**,**D**) microwave power and microwave extraction time; (**E**,**F**) ratio of water to raw material and microwave extraction time, respectively.

The predicted models were presented in three-dimensional (3D) response surface plots and two-dimensional contour plots as shown in Figure 2. Generally, for 2D contour plots, an elliptical contour plot indicates the significant interactions between the variables [35]. And a further understanding was given by 3D response surface plots. In this study, it was clear that the interaction between the microwave power and the ratio of water to raw material (Figure 2B), the microwave power and the extraction time (Figure 2D), and the ratio of water to raw material and the extraction time (Figure 2F) were significant, respectively. Furthermore, the model predicted that the maximum yield (8.05%) could be obtained by the optimal extraction conditions: the microwave power of 415.05 W; the ratio of water to raw material, 51.41 mL/g; and the extraction time of 6.55 min. However, considering the practical operability, the operating conditions were: a power of 415 W; the ratio of 51 mL/g; and the time of 7.0 min. The actual yield of 8.02 ± 0.19% (n = 3) was obtained, which is similar to the predicted value. The results showed that the model for MAE was accurate and adequate in the present study. Furthermore, the MAE method optimized in the present study possessed a higher extraction yield (8.05%) and extremely shorter extraction time (7.0 min) than that of the previously optimized HWE method (extraction yield of 5.46% with the extraction time of 210.0 min) [2]. Indeed, the extraction time of the optimized MAE method was also shorter than that of the previously reported microwave-assisted aqueous two-phase extraction method (extraction time of 20.0 min) [31].

### 2.2. Comparison of Physicochemical Characteristics of CSPs from Cassia Seed Extracted by HWE (CSP-W) and MAE (CSP-M)

#### 2.2.1. Chemical Compositions of CSP-W and CSP-M

The extraction yields of CSPs extracted by HWE (CSP-W) and MAE (CSP-M) were determined as 8.17 ± 0.33% and 8.02 ± 0.19%, respectively, which were a little higher than that of the previous study [11]. Results showed that the extraction yields of CSPs under the optimal extraction conditions of MAE and HWE were almost the same, and the advantage of MAE mainly regarded the reduced extraction time. Therefore, considering the time and temperature (Table 3), MAE could be better than HWE. High carbohydrate contents and low protein contents were observed in CSP-W (80.76 ± 1.19% and 4.80 ± 0.03%) and CSP-M (85.38 ± 1.04% and 5.50 ± 0.12%), respectively. In addition, the total uronic acids content in CSP-W (20.14 ± 0.59%) was significantly (*p* < 0.05) higher than that of CSP-M (18.44 ± 0.67%). The results suggest that different extraction methods had significant effects on the contents of uronic acids in the polysaccharides [29]. Indeed, the low content of uronic acids in CSP-M might be due to the degradation of CSPs under microwave irradiation [21].

#### 2.2.2. Molecular Weights, Intrinsic Viscosities, and Constituent Monosaccharides of CSP-W and CSP-M

**Figure 3:**
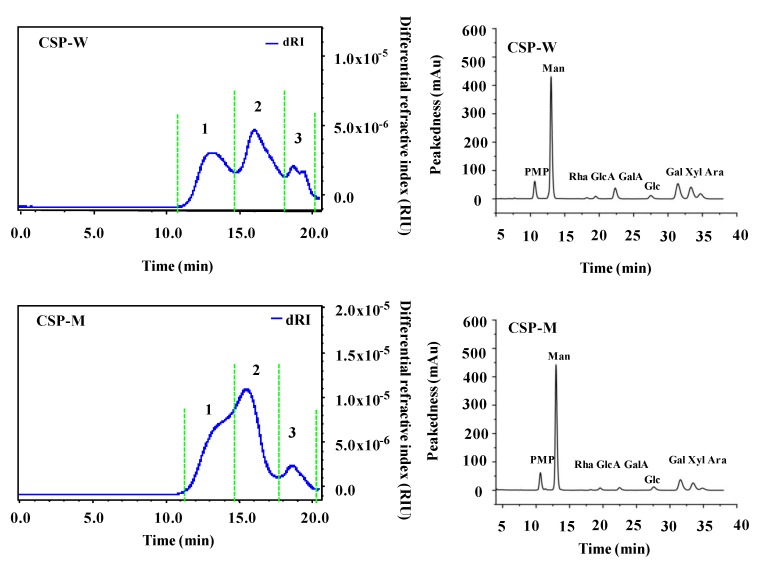
High performance size exclusion chromatograms (left) and high performance liquid chromatography profiles (right) of CSP-W and CSP-M. **CSP-W**, Cassia seed polysaccharides extracted by hot water extraction; **CSP-M**, Cassia seed polysaccharides extracted by microwave-assisted extraction; **PMP**, 1-phenyl-3-methyl-5-pyrazolone; **Man**, mannose; **Rha**, rhamnose; **GlcA**, glucuronic acid; **GalA**, galacturonic acid; **Glc**, glucose; **Gal**, galactose; **Xyl**, xylose; **Ara**, arabinose.

Generally, the activities of polysaccharides are correlated to their molecular weights (*M_w_*) and constituent monosaccharides [36]. When their *M_w_* decrease, some polysaccharides might show decreased intrinsic viscosity and enhanced bioactivities [23,37]. Therefore, these chemical characteristics of CSPs extracted by HWE and WAE were compared. Figure 3 shows the HPSEC-RID chromatograms of the CSPs. Three fractions (Figure 3, fraction 1, 2, and 3) were detected in CSP-W and CSP-M. As shown in Table 4, the *M_w_* of three polysaccharide fractions (fraction 1, fraction 2, and fraction 3) in CSP-W were determined to be 1.337 × 10^6^ Da, 9.838 × 10^4^ Da, and 2.514 × 10^4^ Da, respectively, and the *M_w_* of three fractions (fraction 1, fraction 2, and fraction 3) in CSP-M were determined to be 1.091 × 10^6^ Da, 1.476 × 10^5^ Da, and 3.615 × 10^4^ Da, respectively. Moreover, fraction 1 and fraction 2 were the dominant peaks, which were a little higher than that of the polysaccharides isolated from the seeds of Cassia [11,38,39]. The results indicated that the high *M_w_* fraction (fraction 1) of CSP-W were degraded and converted into lower *M_w_* fractions (fraction 2) during microwave-assisted extraction (Figure 3). Studies have indicated that the *M_w_* of polysaccharides extracted by MAE are lower than that of conventional HWE [23,29]. Meanwhile, the polydispersities of different fractions in CSP-W were 1.562, 1.423, and 1.255, respectively, and that of CSP-M were 1.327, 1.234, and 1.170, respectively. In addition, the activities of polysaccharides such as anti-inflammatory and antioxidant activities are influenced by the intrinsic viscosity [η] [40]. As shown in Table 4, the [η] of CSP-W and CSP-M were observed as 2.81 ± 0.05 dL/g and 2.70 ± 0.04 dL/g, respectively, which is higher than the intrinsic viscosities of Cassia seed polysaccharides in a previous study [39]. The [η] of CSP-M was significantly lower than that of CSP-W, suggesting the intrinsic viscosity can be decreased under the microwave treatment [40].

Furthermore, Figure 3 also shows the HPLC-UV profiles of CSPs. Similar constituent monosaccharides were measured in CSP-W and CSP-M, which were determined as Man, Rha, GlcA, GalA, Glc, Gal, Xyl, and Ara. The major compositional monosaccharides were similar to some previous studies, suggesting that the CSPs obtained in this study contained pectic polysaccharides [10,11]. As shown in Table 4, the molar ratios of Man, Rha, GlcA, GalA, Glc, Gal, Xyl, and Ara in CSP-W and CSP-M were determined to be 2.28:0.07:0.12:0.52:0.17:1.00:0.79:0.29 and 2.88:0.05:0.06:0.14:0.20:1.00:0.72:0.13, respectively. Results suggest that the microwave assisted extraction had no effect on the types of compositional monosaccharides of CSPs, but affected their molar ratios. Previous studies have indicated that extraction methods can affect the molar ratios of compositional monosaccharides [21,30]. Furthermore, the results suggested that galactomannan (GM), homogalacturonan (HG), glucuronoxylan and arabinogalactan (AG II) might exist in CSPs from Cassia seed extracted by HWE and MAE [10,11,31]. Indeed, previous studies have demonstrated that glucuronoxylan and galactomannans exist in the CSPs [17,18,39].

#### 2.2.3. Fourier Transform Infrared Spectra and Degree of Esterification of CSP-W and CSP-M

**Figure 4:**
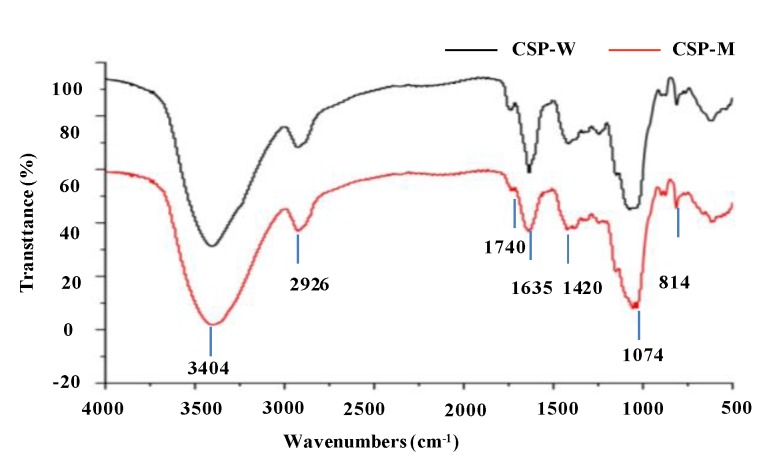
FT-IR spectra of CSP-W and CSP-M. **CSP-W**, Cassia seed polysaccharides extracted by hot water extraction; **CSP-M**, Cassia seed polysaccharides extracted by microwave-assisted extraction.

Fourier transform infrared (FT-IR) spectra between 4000 and 500 cm^−1^ were obtained for the analysis of organic functional groups in CSP-W and CSP-M, and the results are shown in Figure 4. The similar Fourier transform infrared spectra of CSPs indicated that CSP-W and CSP-M have similar structures. In brief, the absorption at 3404 cm^−1^ corresponded to the stretching vibration of the hydroxyl group, while the weak peak at 2926 cm^−1^ corresponded to the stretching vibration of C–H [21,40]. The absorption bands between 1780 cm^−1^ and 1710 cm^−1^ are due to the C=O stretching vibration of the esterified groups [38]. Furthermore, the intense peak that appeared at 1635 cm^−1^ was assigned to the C=O asymmetric stretching of COO^−^, suggesting the existence of uronic acids in CSPs, which was also confirmed by the m-hydroxydiphenyl analysis method and compositional monosaccharide analysis [2,21,41]. In addition, the band at 1420 cm^−1^ was assigned to the C–H stretching vibrations or O–H deformation vibrations [2,42,43]. Typical protein bands at 1651 cm^−1^ and 1555 cm^−1^ were not detected, which indicated the low amount of proteins in CSPs. Results were similar with the low contents of proteins determined by Bradford’s method [42]. Moreover, the band at 814 cm^−1^ corresponded to α-d-galactopyranose [2], which is similar to the constituent monosaccharides of CSPs. Furthermore, the degrees of esterification (DE) of CSPs extracted by HWE and MAE could be obtained by the FT-IR results. Significantly (*p <* 0.05) higher DE values (11.88%) were observed in CSP-W, and lower values (4.70%) in CSP-M. Previous studies have indicated that the low DE might contribute to the relatively high antioxidant activity [44]. Furthermore, results suggest that the MAE method significantly affected the DE of CSPs, and that the low DE value might be caused by these harsh conditions [45,46].

### 2.3. Comparison of Antioxidant Activities of CSPs from Cassia Seed Extracted by HWE and MAE

**Figure 5:**
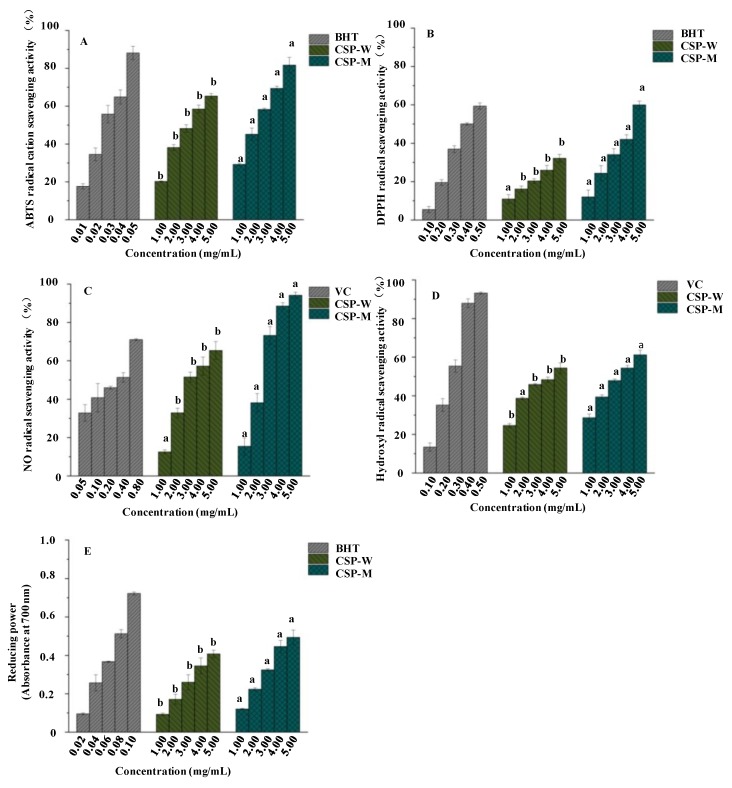
ABTS (**A**), DPPH (**B**), nitric oxide (**C**), and hydroxyl (**D**) radical scavenging activities, and reducing powers (**E**) of CSP-W and CSP-M. **CSP-W**, Cassia seed polysaccharides extracted by hot water extraction; **CSP-M**, Cassia seed polysaccharides extracted by microwave-assisted extraction; The error bars are standard deviations; significant (*p < 0.05*) differences between CSP-W and CSP-M at the same concentration are shown by data bearing different letters (a,b); statistical significances were carried out by an independent sample *t*-test.

Studies have indicated that CSPs from Cassia seed possess remarkable antioxidant activities [2,31]. Therefore, in this study, the in vitro antioxidant activities of CSPs were determined. The ABTS, DPPH, NO, and OH radical scavenging activities, and reducing powers of CSPs are shown in Figure 5, respectively. CSPs exhibited strong in vitro antioxidant activities and different extraction methods could affect the antioxidant activities of CSPs. Briefly, as shown in Figure 5A, CSP-W and CSP-M had substantial scavenging activities for the ABTS radical cation at all tested concentrations in a dose-dependent manner, and the significantly (*p* < 0.05) higher ABTS radical scavenging activities were observed in CSP-M. The MAE could be a good potential method for the extraction of Cassia seed polysaccharides. Additionally, the IC_50_ values of the ABTS radical scavenging activities of CSP-W and CSP-M were determined as 3.04 mg/mL and 2.11 mg/mL, respectively. This shows that CSP-M has a moderate antioxidant activity, even if the IC_50_ value of CSP-M is higher than BHT (a positive control, IC_50_ = 0.025 mg/mL). Moreover, CSP-M showed higher antioxidant activity than that of other polysaccharides [40,47,48,49]. As shown in Figure 5B, the DPPH radical scavenging activities of CSPs also exhibited a dose-dependent manner. CSP-M showed significantly (*p* < 0.05) higher DPPH radical scavenging activities than that of CSP-W. Indeed, the IC_50_ values of CSP-W and CSP-M were determined as 5.83 mg/mL and 4.41 mg/mL, respectively. CSP-M also exhibited stronger DPPH radical scavenging activities than CSP-W, but still lower than the BHT (a positive control, IC_50_ = 0.41 mg/mL). As shown in Figure 5C, CSPs extracted by HWE and MAE also exhibited obvious scavenging activities on NO in a dose-dependent manner. The IC_50_ values of CSP-W and CSP-M were determined as 3.19 mg/mL and 2.10 mg/mL, respectively, which were higher than that of vitamin C (IC_50_ = 0.23 mg/mL). Furthermore, as shown in Figure 5D, CSPs extracted by HWE and MAE also exerted OH scavenging activities. The IC_50_ values of CSP-W and CSP-M were determined as 4.01 mg/mL and 3.16 mg/mL, respectively, which confirmed that CSP-M exhibited stronger OH radical scavenging activities than that of CSP-W. In addition, compared with vitamin C (IC_50_ = 0.23 mg/mL), CSP-M also exhibited moderate OH radical scavenging activities.

Moreover, as shown in Figure 5E, a significantly (*p* < 0.05) higher reducing power was also in CSP-M from 1.0 mg/mL to 5.0 mg/mL, followed by a lower reducing power in CSP-W. Although CSP-M showed a lower reducing power than BHT, while at the concentration of 5.0 mg/mL, its absorbance still reached 0.49 at 700 nm. CSPs could be one of the major contributors toward the antioxidant activities of Cassia seed. In general, the antioxidant activities of polysaccharides are related to their structural features, *M_w_*, and compositional monosaccharides (especially uronic acids) [21,40,50,51]. The presence of electrophilic groups such as keto or aldehyde in acidic polysaccharides could improve the radical scavenging activities [30]. In this study, the higher antioxidant activities (ABTS, DPPH, NO, and OH radical scavenging activities, and reducing power) observed in CSP-M might be partially attributed to its lower *M_w_* and higher content of unmethylated galacturonic acids [4,23]. However, further purification, structural characterization, and in vitro and in vivo antioxidant activities of CSPs are required to reveal their relationships between structure and bioactivity.

## 3. Material and Methods

### 3.1. Samples and Chemicals

Cassia seeds were obtained from a market in Ya’an, China and dried at a temperature of 45 °C for two days. Subsequently, the dried Cassia seeds were ground to pass through a 60 mesh sieve and stored at −20 °C for further analysis.

Trifluoroacetic acid, rhamnose, glucuronic acid, galacturonic acid, mannose, glucose, galactose, xylose, arabinose, m-hydroxydiphenyl, griess reagent, sodium nitroprusside (SNP), 1-phenyl-3-methyl-5-pyrazolone (PMP), 2,2-diphenyl-1-(2,4,6-trinitrophenyl) hydrazyl (DPPH), 2,2′-azino-bis(3-ethylbenzothiazoline-6-sulfonic acid) (ABTS), hydrogen peroxide, vitamin C, and butylated hydroxytoluene (BHT) were purchased from Sigma-Aldrich (St. Louis, MO, USA). All other reagents and chemicals used were of analytical grade.

### 3.2. Extraction of Polysaccharides from Cassia Seed

#### 3.2.1. Hot Water Extraction of CSPs

Hot water extraction (HWE) was performed based on a previously reported method with minor modifications [52]. Briefly, the Cassia seed powders (1.0 g) were first refluxed with 10 mL of 80% (*v*/*v*) ethanol at 80 °C for 2 h to remove most of the small molecules. Then, the polysaccharides from Cassia seed (CSPs) were extracted twice with 30 mL of deionized water at 90 °C for 2 h. Furthermore, four volumes of 95% ethanol (*v*/*v*) were utilized for the precipitation of polysaccharides in the supernatants over night at 4 °C. Finally, the crude polysaccharides extracted from Cassia seed (CSP-W) were freeze dried and stored at −20 °C for further analysis.

#### 3.2.2. Microwave-Assisted Extraction of CSPs

Microwave-assisted extraction (MAE) was performed based on a previously reported method with some modifications [30]. Both the single-factor experimental design and Box-Behnken experimental design were also applied for the optimization of the MAE conditions. Briefly, the Cassia seed powders (1.0 g) were first refluxed with 10 mL of 80% (*v*/*v*) ethanol at 80 °C for 2 h to remove most of the small molecules. Then, the extract residue was extracted with deionized water by MAE (MKJ-J1-3, Qingdao Makewave Microwave Applied Technology Co. Ltd., Shandong, China) and the effects of the microwave power (240, 320, 400, 480, and 560 W; while the extraction time and the ratio of water to raw material were set as 6 min and 50 mL/g, respectively), extraction time (2, 4, 6, 8, and 10 min; while the microwave power and the ratio of water to raw material were set as 400 W and 50 mL/g, respectively), and ratio of water to raw material (30, 40, 50, 60, and 70 mL/g; while the microwave power and the extraction time were set as 400 W and 6 min, respectively) on the yields of CSPs were investigated using a single-factor experimental design. Finally, the crude polysaccharides extracted from Cassia seed (CSP-M) were obtained according to the same treatment processes as described in Section 3.2.1.

A three-level Box-Behnken experimental design (BBD) was applied to further optimize the MAE conditions. The microwave power (X_1_, W), ratio of water to raw material (X_2_, mL/g), and extraction time (X_3_, min) were preferred for the independent variables. The variables and their levels, both the coded and actual values, are presented in Table 1. Experimental data from BBD were explained by the second-order polynomial model as follows [23]:

$$Y=\;A_{0}\;+\;\overset{3}{\underset{i=1}{\sum }}A_{i}X_{i}\;+\;\overset{3}{\underset{i=1}{\sum }}A_{ii}X^{2}{}_{i}\;+\;\overset{2}{\underset{i=1}{\sum }}\overset{3}{\underset{j=i+1}{\sum }}A_{ij}X_{i}X_{j}$$

where Y is the predicted response; *X_i_* and *X_j_* are different variables (*i* ≠ *j*); and *A*_0_, *A_i_*, *A_ii_*, and *A_ij_* are the regression coefficients for intercept, linearity, square, and interaction, respectively.

### 3.3. Characterization of Polysaccharides from Cassia Seed Extracted by HWE and MAE

#### 3.3.1. Chemical Composition Analysis

The phenol-sulfuric acid assay, m-hydroxydiphenyl assay, and Bradford’s assay were utilized for the analysis of the contents of total polysaccharides, uronic acids, and proteins [53,54,55]. In this study, the mixture standard prepared by 50% of mannose, 30% of galactose, and 20% xylose was used to determine the contents of total polysaccharides. The contents of uronic acids and proteins in CSPs were determined with galacturonic acid and bovine serum albumin as the standards, respectively.

#### 3.3.2. Determination of Molecular Weights of CSP-W and CSP-M

The absolute weight-average molecular weights (*M_w_*) and polydispersities (*M_w_*/*M_n_*) of CSPs were measured by high-performance size-exclusion chromatography coupled with a multi-angle laser light scattering and refractive index detector (HPSEC-MALLS-RID), according to our previously reported method [40]. The Astra software (version 7.1.3, Wyatt Technology Co., Santa Barbara, CA, USA) was utilized for data acquisition and analysis. The *M_w_* was calculated by the Zimm method of static light scattering based on the basic light scattering equation. 

#### 3.3.3. Determination of Intrinsic Viscosities of CSP-W and CSP-M

The intrinsic viscosities ([η]) of CSP-M and CSP-W were measured by a modified Ubbelohde viscosity method reported by our previous study [52]. The kinetic energy correlation was assumed to be negligible, and the Huggins and Kraemer equations were used to estimate the value of [η].

#### 3.3.4. Determination of Constituent Monosaccharides of CSP-W and CSP-M

Constituent monosaccharides of CSP-W and CSP-M were measured by high-performance liquid chromatography analysis according to our previously reported method [21]. Briefly, 4 mg of each sample was hydrolyzed with 2.0 M trifluoroacetic acid at 95 °C for 8 h. Then, the hydrolyzates were used for 1-phenyl-3-methyl-5-pyrazolone PMP derivatization. Meanwhile, a standard solution containing Rha, Man, GlcA, GalA, Glc, Gal, Xyl, and Ara was also derivatized by PMP. Finally, an Agilent 1260 series LC system (Agilent Technologies, Palo Alto, CA, USA) coupled with a ZORBAX Eclipse XDB-C18 column (4.6 × 250 mm i.d. 5 µm) and a diode array detector (DAD, Agilent Technologies, Palo Alto, CA, USA) was used to analyze the PMP derivatives.

#### 3.3.5. Fourier Transform Infrared (FT-IR) Spectroscopy Analysis

A previously described method was adopted for the FT-IR spectroscopy analysis of CSPs [29]. The esterification degrees (DE) of CSPs were also determined from the FT-IR spectra according to previous methods [2,38,41]. The determination of DE was based on the band areas at 1780–1710 cm^−1^ (esterified uronic acids) and 1600–1640 cm^−1^ (free uronic acids). DE was calculated according to the equation as follows:

$$\mathrm{DE}\left(\%\right)=\left(\frac{A_{1740}}{A_{1740}+A_{1635}}\right)\times 100$$

### 3.4. Evaluation of Antioxidant Activities of Polysaccharides from Cassia Seed Extracted by HWE and MAE

The ABTS, DPPH, nitric oxide (NO), hydroxyl radical (OH) scavenging activities of CSP-W and CSP-M were determined according to our previously reported methods [30,56,57]. These activities were measured at five different concentrations, and a logarithmic regression curve was established to calculate the IC_50_ values (mg/mL). Both BHT and vitamin C were used as positive controls.

In addition, the reducing power of CSPs was also determined according to our previously reported method [56]. The absorbance was measured at 700 nm. The blank control contained all reagents except the sample. BHT was used as the standard and the reducing power of CSPs was expressed as absorbance at 700 nm.

### 3.5. Statistical Analysis

All experiments were conducted in triplicate, and data were expressed in means ± standard deviations. The obtained data were analyzed by the statistical package of the Design Expert software 8.0.5 (Stat-Ease Inc., Minneapolis, MN, USA). Statistical analysis was performed using Origin 9.0 software (OriginLab Corporation, Northampton, MA, USA). Statistical significances were carried out by an independent-sample t-test. Values of *p* < 0.05 were considered as statistically significant.

## 4. Conclusions

In this study, the optimal extraction conditions of MAE for the extraction of CSPs were obtained by using the response surface methodology. Furthermore, the contents of uronic acids, molecular weights, molar ratios of constituent monosaccharides, and degrees of esterification of CSPs were significantly affected by the MAE and HWE methods. In addition, CSP-M exhibited strong antioxidant activities, which might be partially attributed to its low molecular weight and high content of unmethylated galacturonic acid. Results suggest that the MAE method could be an efficient technique for the extraction of CSPs with high antioxidant activity, and CSPs could be further explored as functional food ingredients.

## Figures and Tables

**Table 1:** Box-Behnken design with independent variables and observed values for microwave-assisted extraction (MAE).

Variable	Levels of Independent Factors			Extraction Yields %
	X_1_ (W)	X_2_ (mL/g)	X_3_ (min)	
**1**	−1 (320)	0 (50)	−1 (4)	6.08
**2**	0 (400)	0 (50)	0 (6)	7.98
**3**	0 (400)	0 (50)	0 (6)	7.89
**4**	1 (480)	0 (50)	1 (8)	7.14
**5**	0 (400)	0 (50)	0 (6)	8.01
**6**	1 (480)	0 (50)	−	6.83
**7**	0 (400)	1 (60)	1 (8)	7.34
**8**	−1 (320)	0 (50)	1 (8)	7.01
**9**	−1 (320)	−1 (40)	0 (6)	6.28
**10**	1 (480)	1 (60)	0 (6)	7.12
**11**	1 (480)	−1 (40)	0 (6)	6.72
**12**	0 (400)	0 (50)	0 (6)	7.96
**13**	−1 (320)	1 (60)	0 (6)	6.37
**14**	0 (400)	0 (50)	0 (6)	8.04
**15**	0 (400)	−1 (40)	−1 (4)	6.38
**16**	0 (400)	1 (60)	−1 (4)	6.19
**17**	0 (400)	−1 (40)	1 (8)	6.60

**X_1_**, microwave power (W); **X_2_**, ratio of water to raw material (mL/g); **X_3_**, extraction time (min).

**Table 2:** Analysis of the variance of the regression equation and coefficients of microwave-assisted extraction.

Source ^a^	Microwave-Assisted Extraction				
	Sum of Square	df ^b^	Mean Square	*F*-Value	*p*-Value ^c^
**Model**	7.90	9	0.88	227.16	<0.0001 **
**X_1_**	0.54	1	0.54	139.36	<0.0001 **
**X_2_**	0.14	1	0.14	35.42	0.0006 **
**X_3_**	0.85	1	0.85	219.73	<0.0001 **
**X_1_X_2_**	0.023	1	0.023	6.02	0.0439 *
**X_1_X_3_**	0.092	1	0.092	23.88	0.0018 **
**X_2_X_3_**	0.22	1	0.22	56.26	0.0001 **
**X_1_^2^**	1.55	1	1.55	401.81	<0.0001 **
**X_2_^2^**	2.33	1	2.33	603.76	<0.0001 **
**X_3_^2^**	1.53	1	1.53	395.76	<0.0001 **
**Residual Error**	0.027	7	3.864 × 10^−3^		
**Lack of Fit**	0.015	3	5.009 × 10^−3^	1.67	0.3099
**Pure Error**	0.012	4	3.006 × 10^−3^		
**Correlation Total**	7.93	16			

*R*^2^ = 0.9966, *R*^2^_adj_ = 0.9922, coefficient of variation = 0.88%, adeq. precision = 40.84; ^a^ **X_1_**, microwave power (W); **X_2_**, ratio of water to raw material (mL/g); **X_3_**, microwave extraction time (min); ^b^ **df**, the degree of freedom; ^c^ *, Significantly different (*p* < 0.05), ** Extremely significantly different (*p* < 0.01).

**Table 3:** Extraction conditions and chemical composition of CSP-W and CSP-M.

Chemical Composition	Samples	
	CSP-W	CSP-M
**Extraction Yields (%)**	8.17 ± 0.33 ^a^	8.02 ±0.19 ^a^
**Extraction Time (min)**	240	7
**Extraction Temperature (°C)**	90	85
**Total Polysaccharides (%)**	80.76 ± 1.19 ^b^	85.38 ± 1.04 ^a^
**Total Uronic Acids (%)**	20.14 ± 0.59 ^a^	18.44 ± 0.67 ^b^
**Degree of Esterification (%)**	11.88± 0.67 ^a^	4.70 ± 0.25 ^b^
**Proteins (%)**	4.80 ± 0.03 ^b^	5.50 ± 0.12 ^a^

**CSP-W**, Cassia seed polysaccharides extracted by hot water extraction; **CSP-M**, Cassia seed polysaccharides extracted by microwave-assisted extraction; Values represent mean ± standard deviation, and superscripts a and b differ significantly (*p* < 0.05) among **CSP-W** and **CSP-M**; Statistical significances were carried out by independent-sample *t*-test.

**Table 4:** Molecular weights (*M_w_*), polydispersities (*M_w_*/*M_n_*), intrinsic viscosities ([η]), and constituent monosaccharides of CSP-W and CSP-M.

	**Samples**	
	**CSP-W**	**CSP-M**
***M_w_* × 10^4^ (Da, Error)**		
**Fraction 1**	133.7 (±1.55%) ^a^	109.1 (±1.09%) ^b^
**Fraction 2**	9.838 (±1.88%) ^b^	14.760 (±2.33%) ^a^
**Fraction 3**	2.514 (±3.66%) ^b^	3.615 (±4.16%) ^a^
***M_w_*** **/*M_n_***		
**Fraction 1**	1.562	1.327
**Fraction 2**	1.423	1.234
**Fraction 3**	1.255	1.170
**[η] (dL/g)**	2.81 ± 0.05 ^a^	2.70 ± 0.04 ^b^
**Monosaccharide Compositions (Molar Ratio)**		
**Mannose**	2.28	2.88
**Rhamnose**	0.07	0.05
**Glucuronic Acid**	0.12	0.06
**Galacturonic Acid**	0.52	0.14
**Glucose**	0.17	0.20
**Galactose**	1.00	1.00
**Xylose**	0.79	0.72
**Arabinose**	0.29	0.13

**CSP-W**, Cassia seed polysaccharides extracted by hot water extraction; **CSP-M**, Cassia seed polysaccharides extracted by microwave-assisted extraction; Values represent mean ± standard deviation, and superscripts a and b differ significantly (*p* < 0.05) among **CSP-W** and **CSP-M**; Statistical significances were carried out by an independent-sample *t*-test.
